# Intracellular DNA sensing by neutrophils and amplification of the innate immune response

**DOI:** 10.3389/fimmu.2023.1208137

**Published:** 2023-07-07

**Authors:** Arun K. Mankan, Paulina Czajka-Francuz, Maria Prendes, Sriram Ramanan, Marcin Koziej, Laura Vidal, Kamal S. Saini

**Affiliations:** ^1^ Fortrea, Inc., Durham, NC, United States; ^2^ Labcorp Drug Development Inc., Princeton, NJ, United States; ^3^ Addenbrooke’s Hospital, Cambridge University Hospitals NHS Foundation Trust, Cambridge, United Kingdom

**Keywords:** neutrophils, DNA sensing pathways, AIM2, SOX2, cGAS, Cancer

## Abstract

As the first responders, neutrophils lead the innate immune response to infectious pathogens and inflammation inducing agents. The well-established pathogen neutralizing strategies employed by neutrophils are phagocytosis, the action of microbicide granules, the production of ROS, and the secretion of neutrophil extracellular traps (NETs). Only recently, the ability of neutrophils to sense and respond to pathogen-associated molecular patterns is being appreciated. This review brings together the current information about the intracellular recognition of DNA by neutrophils and proposes models of signal amplification in immune response. Finally, the clinical relevance of DNA sensing by neutrophils in infectious and non-infectious diseases including malignancy are also discussed.

## 1 Introduction

As part of the first line of protection provided by the innate immune system, neutrophils play a key role in the early immune response. Neutrophils are essential for defense against not only pathogens, such as fungi and bacteria, but they are also the first responders at the site of acute injury or acute inflammation (1, 2).

A defining feature of the pathogen-eliminating mechanism of neutrophils is phagocytosis. Over the last several decades, studies have finely dissected the controlled process by which neutrophilic phagosomes are formed. The phagosome is a distinctive organelle arising from the invagination of the plasma membrane which results in the formation of a vacuole-like structure, thereby completely encircling the engulfed pathogen. Within the phagosome, several simultaneously or consecutively occurring processes take place, which lead to the rapid neutralization of the pathogen. Scientific consensus is that the active processes within neutrophilic phagosomes include the production of NADPH oxidase-derived reactive oxygen species (ROS), the delivery of microbicidal proteins from the pre-formed granule, the movement of ions across the phagosomal membrane and the dynamic alteration of the phagosomal pH (3–5). In fact, the pre-formed granules in mature neutrophils consist of subsets of different granules, like azurophilic, specific, gelatinase and lysosomal granules, which differ not only in their biochemical characteristics but also in their primary functions and participate in all known neutrophil antimicrobial effector activities (5–7). In contrast, the NADPH oxidase complex is responsible for the generation of the superoxide molecule that acts upon the myeloperoxidase enzyme to generate biologically toxic substances, such as hypochlorite. Although, other immune cells like macrophages also phagocytose pathogens, neutrophils are inherently more powerful at this activity by at least 1-2 orders of magnitude (1). A key point to note is that while pathogens can stay in the macrophages latently, neutrophils are rarely used as a resting point by pathogens (1). This implies that the end result of phagocytosis by neutrophils is the complete destruction of the pathogens. And thus, while multiple studies have highlighted the importance of phagocytosis by neutrophils, very few of them have clearly clarified what occurs once the pathogen is killed within the phagosome (3, 8). The resolution of the phagosome with the disintegration of the pathogenic molecular motifs and potential re-integration of the broken-down molecules within the neutrophilic biochemical processes, or the disposal of inactivated foreign molecules, remains largely unexplored. More specifically, the fate of so-called pathogen-associated molecular patterns (PAMPs) including, DNA, RNA, mRNA or proteins originating from the pathogen, still remains an unsolved mystery. Although speculative, one can foresee the possibility that these PAMPs are recognized by intracellular sensors present within neutrophils. The resulting activation of the various signaling cascades and the release of cytokines and chemokines by neutrophils has a direct implication in the resolution of the infection or inflammation. It should be stressed that post phagocytosis, PAMPs are not only present intracellularly in neutrophils, but neutrophils may also present the digested pathogenic protein particles as antigens to the adaptive immune system, thereby ensuring that the immune response against the invading pathogen is further prolonged, as well as amplified (9). This aspect of the amplification of the immune response will be discussed later in this review.

Apart from phagocytosis, formation of NETs is an unique phenomenon observed amongst few immune cells including neutrophils. NETs are composed of web-like decondensed chromatin fragments, which are coated with histones and antimicrobial proteins (10–13). Interestingly, NETs are released only by activated neutrophils, clearly indicating the need for a neutrophil-activating signal (12). Once activated, a sequential program is triggered which involves the permeabilization of the plasma membrane, the disassembly of the cytoskeleton and nuclear envelope, the decondensation of chromatin, and the assembly of the antimicrobial proteins onto the surface of the chromatin (12). NETosis results in the extrusion of both nuclear and mitochondrial DNA (14). While initial experimental results suggested that the primary function of NETs is to trap and neutralize pathogens, currently it is well accepted that NETs also modulate functions of other immune cell, promote thrombosis, tumorigenesis, as well as tumor metastasis (11, 13).

## 2 Intracellular DNA signaling - our understanding so far

The discovery of the role for the first Toll-like receptor (TLR) in providing immunity against fungal and gram-positive bacterial infections, initiated a whole gamut of studies investigating different pattern recognition receptors (PRRs) (15). Starting with the identification of multiple TLRs on the cellular surface of immune cells, subsequent studies identified endosomal/cytoplasmic PRRs. In line with the identification of different PAMPs present within pathogenic organisms, soon their corresponding PRRs were subsequently identified. A widespread group of PAMPs present in all living organisms are the nucleic acids including DNA, RNA, and RNA : DNA hybrids. All these nucleic acids are either localized to certain organelles in the cell, or otherwise subjected to a very tightly controlled transport, thereby limiting the inappropriate exposure to corresponding intracellular sensors. However, under some circumstances, these nucleic acids present themselves in the cytosol or the endosomes, potentially resulting in their recognition as danger signals by PRRs. PRRs that have been implicated in the detection of RNA and DNA derived from both, pathogens and hosts, include Toll-like receptors (TLRs), RIG-I-like receptors (RLRs), absent in melanoma 2 (AIM2)-like receptors (ALRs), and NOD-like receptors (NLRs) (16, 17). This review is primarily limited to elucidating the current knowledge about intracellular recognition of DNA by cytosolic PRRs, with a further narrowed focus about the role of such DNA sensors in neutrophils.

In the last two decades we have uncovered several endosomal/cytosolic DNA sensors, like Toll-like receptor 9 (TLR9), AIM2, cyclic GMP-AMP synthase (cGAS), stimulator of interferon genes (STING), interferon gamma-inducible 16 (IFI16), DEAH- and DEAD-box helicases DHX9, DHX36, DDX41, and RNA polymerase III (16, 18). Broadly speaking, the detection of DNA by these receptors results in cytokine production and, in some cases, initiates an inflammatory and lytic form of cell death called pyroptosis. While a majority of these sensors like TLR9, cGAS, DAI, DEAH- and DEAD-box helicases DHX9, DHX36, DDX41, and RNA polymerase III induce the production of type I IFNs, activation of AIM2 results in the secretion of IL-1β.

**Figure 1:**
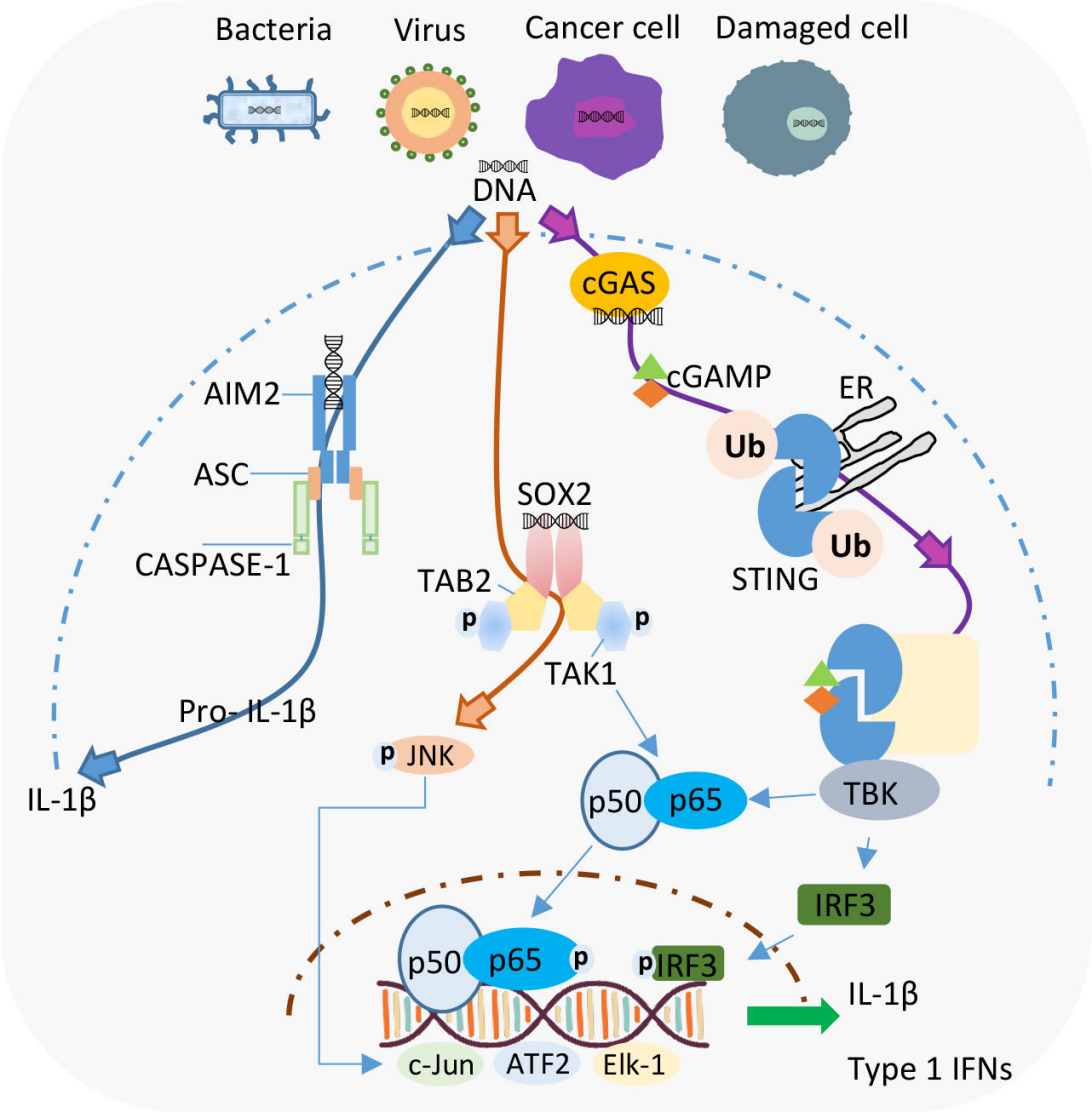
Intracellular DNA recognition by different cytosolic DNA sensors in Neutrophils. Current evidence shows the existence of AIM2 and Sox2 as DNA sensors in neutrophils. cGAS-STING is a well-established cytosolic DNA sensor but the existence of this pathway in neutrophils needs to be further explored.

Despite the long list of reported DNA sensors, currently AIM2 and cGAS are considered as the primary detectors of cytosolic DNA. The AIM2 protein belongs to the family of NLR proteins called inflammasomes, and functions as a molecular platform where caspase-1 and apoptosis-associated speck-like protein (ASC) come together. The subsequent interactions between these proteins results in the maturation and release of IL-1β/IL-18, and gasdermin D (GSDMD)-mediated pyroptosis (19). On the contrary, pathogenic DNA detected by cGAS induces the synthesis of cGAMP (20). cGAMP, functioning as a second messenger, associates with- and activates stimulator of IFN genes (STING). STING has been implicated as a central player in DNA-induced type I IFN production (**Figure 1**). Readers are encouraged to check the innumerable reviews that have put together all the literature about the signaling pathways, molecular regulation, and the role of these two DNA sensors in several diseases (21–23).

### 2.1 Intracellular DNA sensing in neutrophils

Our understanding of the role of neutrophils within the innate immune system is based primarily on their function as a phagocytic factory and their involvement in the phenomenon of NETosis. For several decades, the focus of many studies was on finding out how exactly neutrophils phagocytose pathogens and the microbicidal aspect of the phagosome. Despite it being a well-controlled process, phagocytosis and NETosis involve physical modifications of the neutrophil’s molecular material and they appear as rather primitive mechanisms for neutralizing pathogens; especially when compared with the elegant signaling pathways and cascades that have been identified in other members of the innate immune system, such as macrophages or dendritic cells and in cells belonging to the adaptive immune system. Recently, there has been an attempt to revisit the role of neutrophils in the recognition of PAMPs, including intracellular DNA. In the subsequent section of this review, we will investigate how neutrophils could potentially sense and respond to cytosolic DNA.

#### 2.1.1 Expression of molecular components essential for the sensing of intracellular DNA in neutrophils

For a long time, neutrophils were considered as terminally differentiated, short lived cells with a latent condensed nucleus and pre-formed proteins ready to identify and kill pathogens. The ability of neutrophils to dynamically respond to danger by up- or down-regulating genes in a cell-intrinsic manner was not clear. In fact, one study exploring the changes in neutrophils exposed to *S.aureus* showed minimal changes in protein expression *per se*, but revealed that about one third of the phospo-proteome was altered (3). However, recent data, proving that neutrophils are fully capable of extensive and rapid changes in gene expression upon activation, forces us to revisit this idea of neutrophils as immune cells with merely pre- determined processes. Indeed, in response to neutrophilic stimulation with live/opsonized *E.coli* or soluble LPS, the expression of multiple groups of genes was altered (24). This modified expression upon stimulation ranged from genes involved in the classical response to inflammation/infection (such as members of the FOS, JUN, and NF-κB families), to regulators and mediators of gene expression (i.e. RNA polymerase II transcription factors and protein chaperones. These and other subsequent data now reveal that neutrophils also possess the plasticity to adjust their response to different pathogens (24, 25).

For us to appreciate the relevance of the intracellular DNA sensors in neutrophils, we first need to confirm the presence of at least some of the known components of these signaling pathways in these cells. We know that neutrophils express all TLRs from 1-10 except TLR3 (26). Of these, TLR9 recognizes the unmethylated cytosine-phosphate-guanosine (CpG) form of DNA, which is commonly found in bacterial, viral, fungal, and parasite genomes. In brief, on one end TLR9 functions *via* the MyD88 protein to activate the IKK-NFκB pathway, and on the other end it acts through the IRF-IFN pathway, resulting in the secretion of IFN and IL-1β. Although TLR9 is expressed in neutrophils, some early studies reported that TLR9 is not required for nucleosome-induced neutrophil activation (27). However, recent studies have confirmed, that mitochondrial DNA, released as a result of cellular trauma, can activate neutrophils in a TLR9-dependent manner (28).

The presence of AIM2 in murine and human neutrophils has been well established since some time (29, 30). Despite this, only recently a functional role for AIM2 protein in detecting cytosolic DNA was established (31). For a long time, it was assumed that some of the enzymatic components of the neutrophilic granules, like neutrophil elastase and proteinase 3, were essential for the maturation and secretion of IL-1β by neutrophils. Based on genetic models, the absolute requirement for these enzymes in the production of IL-1β has now been disproved. In fact, several studies have now demonstrated that, almost all essential components required for the activation of different inflammasome pathways are expressed in neutrophils (29, 30, 32). More specifically, it has been shown that neutrophils express functionally active caspase-1 and NLRP3 protein, and that they can induce and secrete mature IL-1β in a dose-dependent manner. Thus, all components essential for activation of AIM2 pathway are present in neutrophils.

**Figure 2:**
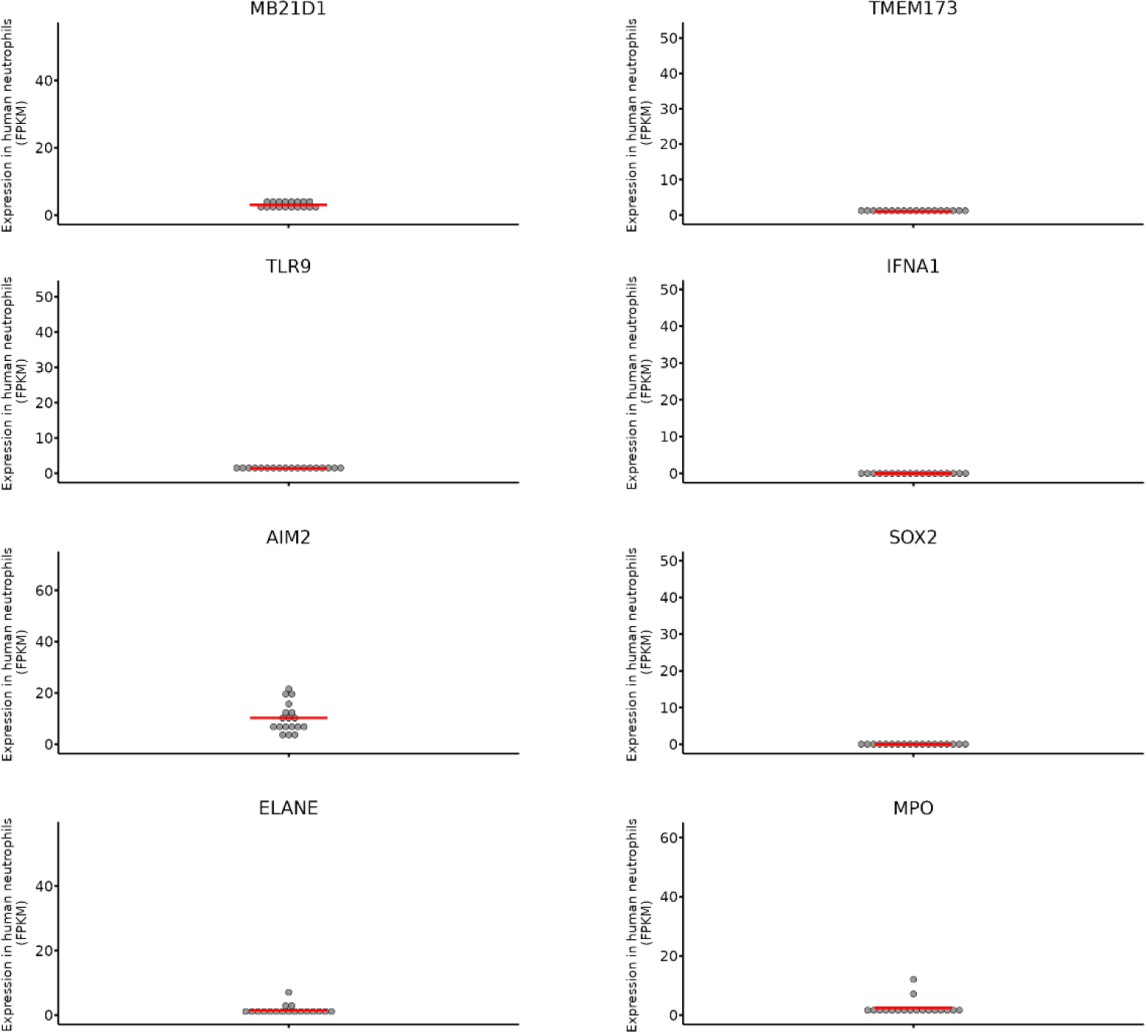
The expression of different genes known to be essential for intracellular DNA sensing were checked on the Neutgx portal from NIH (https://neutgx.niaid.nih.gov/) (34). Each dot represents expression of the gene in one biological replicate. Expression is measured as FPKM (fragments per kilobase of transcript per million fragments mapped). Established neutrophil specific genes *ELANE* (Neutrophil elastase) and *MPO* (Myeloperoxidase) are used as control. *MB21D1* and *TMEM173* are the names for human *CGAS* and *STING* genes. It should be noted that expression of *CGAS* and *STING* genes in neutrophils is in the same range as that of *SOX2*.

More recently, Xia, Wang and colleagues identified a completely new neutrophil-specific intracellular DNA sensing protein called Sox2 (33). Before this report came out, Sox2 was known as a transcription factor with a key role during fetal development. Gene expression data revealed that Sox2 is constitutively expressed in the cytoplasm of peripheral neutrophils derived from both, mice and humans. Neutrophil-specific deletion of *SOX2* resulted in increased susceptibility of mice to infection with listeria, and it validated the important role for this protein in neutrophils. The authors showed that binding of pathogenic DNA with Sox2 triggered Sox2 dimerization *via* its GBH domain resulting in the activation of TAB2-TAK1 complex resulting in the translocation of NF-κB and AP-1 transcription factors and the production of TNFα, IL-6 and IL-1β pro-inflammatory cytokines. An important point that should be noted is that in this study the authors demonstrated that neutrophils have low expression of STING and undetectable expression of cGAS and that the Sox2-mediated activation of neutrophils was independent of STING or cGAS. We need to reconcile this data with the data from Neutgx portal mentioned below wherein one can conclude that the expression of *SOX2*, *CGAS* and *STING* are in the similar range (**Figure 2**). Interestingly, Sox2 is not expressed in T cells, B cells, natural killer cells, dendritic cells or macrophages and this raises an open-ended question about, why only neutrophils express Sox2 as an exclusive intracellular DNA sensor (33). Further studies are required to understand whether Sox2 is associated with any other aspect of neutrophil function, such as, phagocytosis or NETosis (35, 36).

Due to the absence of additional experimental proof on the expression of all cGAS-STING pathway components in neutrophils, for the purpose of this review, we verified the gene expression profiles provided on the Neutgx portal from NIH (https://neutgx.niaid.nih.gov/) (34). The neutrophil gene expression data offered by NeutGX was obtained from bulk RNA sequencing (RNA-seq) experiments performed on highly pure neutrophils obtained from the peripheral blood of healthy human volunteers (34). As such it provides unbiased data for the expression of different classes of genes in neutrophils. As shown in **Figure 2** both *STING* (human gene *TMEM173*) and *CGAS* (*MB21D1*) are indeed expressed by human neutrophils. The presence of measurable levels of mRNA for these targets in neutrophils indicates that *CGAS* and *STING* could be translated into proteins within human neutrophils allowing a potential functional role for these sensors in recognizing intracellular DNA. The comparison with the expression levels of neutrophil elastase and myeloperoxidase genes (*ELANE*; *MPO*), characterized in the Neutgx portal as neutrophil specific genes, enables an estimate of *CGAS* and *STING* gene expression level in neutrophils.

As described above, the activation of most intracellular DNA sensors results in the secretion of type 1 IFN. The exception to this rule is AIM2, which when activated promotes the secretion of IL-1β. It is well established, that amongst immune cells, dendritic cells (conventional or plasmacytoid) and macrophages are largely responsible for producing type 1 IFNs. The hint that neutrophils can produce also type 1 IFNs, came indirectly from studies showing the production of these cytokines in disease models where dendritic cells were depleted. Current evidence has, in fact, confirmed the ability of neutrophils to secrete type 1 IFNs in response to poly I:C, free chromatin, mitochondrial DNA, and of course, in response to genetic material from pathogenic organisms (32). In short, it is clear that neutrophils express all known molecular components necessary for recognition of cytosolic DNA.

#### 2.1.2 The potential sources of cytosolic DNA in neutrophils

The question of how genetic material present within the nucleus and barred from the cytosol by the nuclear membrane reaches the cytosol, is a puzzle that still needs to be solved. With respect to neutrophils, there are several potential mechanisms that could explain for the presence of DNA in the cytosol of these cells.

a) Phagocytosis of the whole pathogen including its genetic material. As described in the introduction, while the process of phagocytosis itself has been well studied, what happens to the pathogenic molecular components, once they have been digested by the contents of the neutrophilic granules, has been largely unexplored. It is, therefore, conceivable that the genetic material of the pathogen could be potentially exposed directly to the cytosolic sensors in neutrophils.

b) Ingestion of free floating pathogenic genetic material. At the very initial stages of the infection, during the interaction with the ectodermal/endodermal cells, pathogens may not remain intact and may disintegrate. Furthermore, the cellular enzymes released at the site of infection could degrade the pathogen *in situ*, thereby releasing its genetic material. This free-floating genetic material once ingested by the neutrophil could be presented to the cytosolic nucleic acid sensors (37).

c) Release of mitochondrial DNA. The mitochondrial DNA is known to be released either because of cellular injury or during the formation of the NETs. We now know that mitochondrial DNA is also an inducer of the TLR9-dependent signaling cascades (28, 38, 39). The latest evidence in this regard has revealed that mtDNA can indeed also activate the cGAS-STING pathway (40). Whether the same is true in neutrophils, still needs to be established.

d) Horizontal transfer of genetic material between neutrophils. Experimental evidence shows that neutrophils can communicate with each other using attractants, which once secreted, helps them to bind to cell surface–expressed G protein–coupled receptors (GPCRs) on neighboring cells. This strategy enables the neutrophils to launch a coordinated effort in their hunt for pathogens (41). The neutrophil-cluster formation acts as a physical barrier that limits the spread of the pathogen. However, what remains to be explored is whether PAMPs resulting from neutrophil phagocytosis are potentially transferred to the surrounding naïve neutrophils. In this case, one neutrophil becomes the direct responder to the pathogen, while another neutrophil acts as the receiver of the PAMP element(s) like DNA. As such the horizontal transfer of intracellular DNA could further amplify the signaling cascades resulting in an enhanced concentration of cytokines and chemokines at the point of infection.

#### 2.1.3 The downstream consequences of intracellular DNA sensing by neutrophils

Neutrophils can recruit, interact with and activate other immune cells by secreting cytokines, chemokines, and proteases that can then modulate the immune response (42, 43). Be it at the site of initial insult or distant lymph nodes, neutrophils can stimulate macrophages, induce differentiation of monocytes to monocyte-derived DCs, promote recruitment and maturation of classical antigen presenting cells such as NK and conventional DC cells and drive plasmacytoid DC recruitment resulting in interferon production (1, 44). This modulation of DC activity indirectly has an impact on the T cell function. However, neutrophils also directly induce non-specific T cells by secreting the chemokines that attract T cells to the site of the inflammation. Furthermore, neutrophil depletion models have revealed that neutrophils can also influence the Th cell polarization and affect the balance between the Th1 and Th2 cells (42, 45). The displacement of neutrophils to distant lymph nodes or even to spleen especially while still carrying elements of the pathogen due to ineffective elimination of pathogen, can also result in the activation of B cells. This activation of the B cells results from the secreted B-cell activating factor of the tumor necrosis family (BAFF) and a proliferation-inducing ligand (APRIL) and promotes B cell expansion, plasma cell differentiation and the increased production of antibodies (1, 42, 45). Indeed, experimental evidence has demonstrated a link between neutrophilic DNA, NETs and activation of adjacent macrophages wherein decondensed DNA present in NETs resulted in the activation of the macrophagic cytosolic cGAS-STING pathway (46). This transfer of activating signals from neutrophils to macrophages ultimately results in the amplification of the initial immune response.

When it comes to cancers, within the tumor micro environment (TME) the interaction of the invading neutrophils with the surrounding immune cells depends on the phenotype of neutrophils. We now know that neutrophils can differentiate into two phenotypes with different properties: N1 –phenotype with pro-inflammatory properties and N2 phenotype showing immunosuppressive profile. N1 pro-inflammatory neutrophils are activated by type I interferons, inhibit angiogenesis and are able to eliminate pathogens *via* antibody-dependent cellular cytotoxicity (ADCC) and phagocytosis. These cells show increased NADPH oxidase activity which leads to the production of reactive oxygen species (47). Immune profile of N1 cells is characterized by the secretion of high levels of TNFα, CCL3, intercellular adhesion molecule 1 (ICAM-1), and low levels of arginase. N2 phenotype neutrophils induce immunosuppression *via* release of CXCL1, MMP9, VEGF, and TNFα (48, 49). Moreover, these neutrophils act *via* releasing ROS and nitric oxide (NO), which increase DNA instability. N2 neutrophils can inhibit T cell proliferation *via* expression of arginase 1 and induce T cell apoptosis *via* NO production (50).

Having established the effect of activated neutrophils on other immune cell types we can next focus on the consequences of effector cytokines secreted because of intracellular DNA sensing i.e., IL-1β and type I IFNs. IL-1β itself has been shown to enhance antigen-driven response in both CD4 and CD8 T cells, support the expansion and activation of specific Th1, Th2, Th17 and Granzyme B+ CD8 T cells *in vivo* and for naïve CD4+ T cells to overcome Treg-mediated inhibition and memory CD4+ T cells to acquire a fully functional memory phenotype (51).

With regards to type I IFN, meta-analysis of experimental data has revealed that the outcome of the type I IFN stimulation on immune cell activation is largely context dependent and does not always follow a linear “cause-effect” relationship. Type I IFN i.e., IFNα/β have effects on both the innate and adaptive cellular immune response and affect myeloid cells, B cells T cells and NK cells (52). For example, IFNα/β has an activating effect on immature committed DCs, enhancing the expression of MHC molecules and co-stimulatory molecules such as CD80 and CD 86, which is associated with an increased ability to stimulate T cells (53). IFNα/β also promote the migration of DCs to the lymph nodes. DCs themselves are potent producers of IL-12 which is required for driving T helper type responses during certain infections and promoting the production of IFNγ by T and NK cells (52).

## 3 Amplification of the innate immune response-role for neutrophils

**Figure 3:**
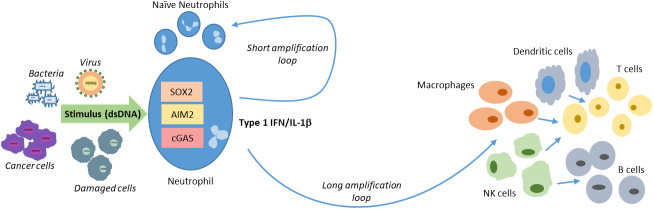
Short and long amplification loops of immune cell activation. At the site of the initial insult neutrophils are activated by directly engaging with the pathogen. These can then activate the subsequent waves of swarming neutrophils creating a short loop. Cytokines and chemokines secreted by the neutrophils further activate immune cells in the milieu there by amplifying the immune response by the longer loop.

As the first responders, neutrophils initiate the immune response to the primary insult. The outcome of the interaction between the pathogen and the neutrophil will depend on several factors. For example, depending on whether the pathogen is wholly phagocytosed or some specific element of the pathogen is phagocytosed or if only specific PAMP(s) bind and activate PRR(s), activated neutrophils will secrete different cytokines and chemokines. Although, in comparison with DCs or macrophages, in general, neutrophils do secrete only a small amount of cytokines, the presence of massive numbers of neutrophils in the blood and at the site of insult, ensures an accumulative stronger cytokine response. It is conceivable that the first wave of neutrophils, having engaged with the injurious element, start secreting cytokines, which could in turn activate the subsequent waves of neutrophils. We use the term short amplification loop to describe this local activation of naïve neutrophils. Depending on the time since the initial insult and the efficacy of the initial response, other immune cells such as monocytes, macrophages, DCs, NK cells, T cells and B cells are directed towards and can be stimulated by the secretome of the activated neutrophils. Keeping in mind that in response to activation, macrophages and DCs secrete cytokines several fold higher than neutrophils, the immune response attains a logarithmic scale. This activation of other immune cells and enhanced secretion of cytokines can be considered as the long loop of immune response amplification (**Figure 3**). While these short and long amplification loops can certainly be features of other signaling pathways activated by distinct PAMPs as part of immune responses, couple of unique factors i.e., a) that neutrophils act as the first responders; b) their relatively higher numbers and c) the secretion of potent cytokines like IL-1β and type I IFNs upon recognition of intracellular DNA, attests to the role of neutrophils as central players in this phenomenon.

## 4 Clinical implications

In keeping with its key role in the immune system, neutrophils have been implicated in many different diseases including infectious diseases, acute and chronic inflammatory conditions, autoimmune diseases as well as cancers. In this section, we list some examples of potential clinical relevance for intracellular DNA sensing by neutrophils.

As the prototypic member of the cytosolic DNA sensors, AIM2 is involved in the detection of genetic material from many different bacteria and viruses. Examples of some of the bacteria are *Mycobacterium tuberculosis, Porphyromonas gingivalis, Streptococcus pneumoniae, Chlamydia* muridarum. In addition, viruses such as *Vaccinia virus*, *Human papillomaviruses*, *Hepatitis B virus* and West Nile virus (RNA virus) also activate the AIM2 pathway (54). Similarly, a role for cGAS-STING pathway has also been identified in multiple infectious diseases. Current evidence indicates that this pathway is activated in response to bacteria such as *Listeria monocytogenes, Staphylococcus aureus. Streptococcus pneumoniae, Brucella abortus, Pseudomonas aeruginosa* and viruses including vaccinia virus, Kaposi’s sarcoma-associated herpesvirus and herpes simplex virus (55, 56). Interesting, AIM2, Sox2 and cGAS have all been shown to detect DNA from *Listeria monocytogenes*. While there is ample evidence that supports a role for neutrophils in infection by *Listeria monocytogenes, S. aureus and S. pneumoniae*, how effective neutrophils are in response to viral infections is still not clear (57, 58). As such, whether activation (or deficiency) of AIM2/Sox2/cGAS-STING pathway in neutrophils plays a direct role in the immune response against these pathogens needs to be further explored.

Psoriasis that manifests mainly as skin lesions and extracutaneous comorbidities serves as a classic example of a chronic, immune-mediated disease. In psoriasis, loss of immune homeostasis results in the overstimulation of several different cells including neutrophils, dendritic cells, T cells, keratinocytes, fibroblasts, mast cells, and melanocytes (59). In fact, Munro’s microabscesses that are considered as one of the major histopathological hallmarks of psoriasis are vesicles filled with neutrophils (60). Neutrophilic granules, ROS production and release of NETs have all been found to play a role in psoriasis and presence of MPO and NETs in skin plaques is positively correlated with the severity of the disease (59, 61). In contrast, a reduction in circulating neutrophils by drug induced agranulocytosis or by using the granulocyte and monocyte adsorption (GMA) is accompanied by regression of psoriatic plaque and abscess development (62, 63). The role for activated neutrophils is not just limited to the site of the lesion but it also results in the stimulation of myeloid and plasmacytoid DCs (59). Additionally, the activated neutrophils and mast cells present in the psoriatic skin secrete IL-17 (64). This secreted IL-17 on one hand, acts on keratinocytes directly, and on the other hand, induces the production of both G-CSF and subsequently neutrophils (65). Thus, psoriasis serves as a classic example for the short and long amplification loops resulting from the activation of neutrophils (**Figure 3**). More recently, experimental evidence has shown that abundant dsDNA fragments are present in the upper epidermal layers of psoriatic, but not in the unaffected skin areas (66). One hypothesis suggests that fragments of genomic dsDNA (resulting from skin surface perturbation) present in the epidermis trigger skin inflammation and keratinocyte hyperproliferation (66). Defective cellular differentiation results in parakeratosis i.e., excessive production and abnormal maturation of keratin. Denucleation of the immature keratinocytes leads to the release of genomic DNA fragments in the psoriatic lesions which in turn induce pro-inflammatory cytokines. A role for both cGAS-STING and AIM2 has been observed in the recognition of dsDNA in keratinocytes (67, 68). While whether neutrophils are directly responsible for recognizing the dsDNA in psoriasis and any consequences of this needs to be further explored, it is now clear that neutrophils do play a key role in psoriasis and that the vicious inflammatory cycle resulting from the release of dsDNA in psoriatic lesions should be explored as potential therapeutic target.

Inflammatory Bowel Disease especially, Ulcerative Colitis (UC) serves as a good case of a chronic inflammatory condition where we have sufficient evidence for the contribution of neutrophils in its pathogenesis. UC is a disease of unknown etiology characterized by inflammation of the mucosa and sub-mucosa of the colon and rectum lining (69). Clinically, the chronic inflammation results in ulcers with resultant abdominal pain, weight loss, fever, diarrhea mixed with blood and anemia. UC patients present with a high neutrophil to lymphocyte ratio (70). At the tissue level, one recurrent feature observed in UC is the accumulation of neutrophils in the inflamed intestinal mucosa. As expected, Neutrophilic myeloperoxidase and the NETs have been identified as the main culprits responsible for causing tissue inflammation in UC (69, 71, 72). Therapeutically, decreasing the number of neutrophils either by using steroids or by GMA has turned out to be a promising strategy for reversing the disease progression in UC (73, 74). Recent evidence has also pointed out a role for intracellular DNA sensing and secreted type 1 IFNs in UC (75). Enhanced expression of both AIM2 and cGAS, in a cell type-restricted pattern, was detected in active UC tissue. However, so far, no direct link between intracellular DNA sensing by neutrophils and UC has been established.

Finally, a causative role for neutrophils itself in tumorigenesis is highlighted by the observation that solid tumors infiltrated by large number of these cells or that a higher neutrophil-to-lymphocyte ratio (NLR) in peripheral blood results in poor clinical outcome (76, 77). However, as described above, the phenotype of the neutrophils present in the TME also influences whether tumor associated neutrophils will support an anti-tumor response or have a pro-tumor effect. It is not surprising that several clinical trials targeting neutrophils have been launched for Breast cancer, Hepatocellular carcinoma, Colorectal neoplasms amongst others (76, 77).

Cancer cells, especially those that have advanced dysmorphic features with unstable genomes resulting in chromosomal instability (CIN), present DNA in the cytosol. Furthermore, the oxidative stress that malignant cells are exposed to results in mitochondrial dysfunction and release of mtDNA. Other sources of cytosolic DNA in cancer cells include the leakage of genetic material during cellular senescence (56, 58).

AIM2 has been implicated in the pathogenesis of tumors like colon cancer, hepatic cancer, cutaneous squamous cell carcinoma and endometrial carcinoma. Like several other oncogenic proteins, AIM2 can, in a context dependent manner either promote or inhibit carcinogenesis (78). AIM2 can also recognize DNA released a result of ionizing radiation and/or use of chemotherapeutic drugs (79).

Sox2 plays a role during all stages of carcinogenesis including cancer cell proliferation, migration, invasion, and metastasis (80, 81). Sox2 signaling involves multiple different signaling pathways such as EGFR, SHH, HIPPO, WNT/β-Catenin, and TGF-β/Smads signaling pathways and results in enhanced proliferation, survival, and tumorigenesis. Indeed, Sox2 amplification or overexpression is a frequently occurring event, for example in Breast, Colorectal, Esophageal, Liver, Lung adenocarcinoma, Prostate and is associated with advanced stages of tumorigenesis, poor prognosis, and drug resistance, making Sox2 a target for anti-cancer therapies (82, 83).

We now know that the cytosolic DNA in the cancer cell themselves or within the immune cells present in the TME can be detected by cGAS (56). From a tumorigenesis standpoint, activation of cGAS-STING pathway and the subsequent release of pro-inflammatory cytokines has two major consequences. Firstly, it prevents the early neoplastic progression and secondly it promotes the recruitment of effector immune cells (56). Within the TME, it has been proposed that the cytosolic DNA in cancer cells can be transmitted to adjacent antigen presenting cells like dendritic cells. cGAMP, formed as a result of the binding of dsDNA to cGAS in the cancer cell, can itself be transferred to the adjacent cells in TME by moving through the gap junctions or aided by tumor cell-derived exosomes (56, 58). Finally, a role for this DNA sensing pathway has been identified in the cancer metastasis, autophagy and response to the DNA damaging therapies (56).

However, for the purpose of this review, it should be mentioned that is has, so far, not been possible to study anti-cancer drugs targeting AIM2, Sox2 or cGAS-STING pathway exclusively in neutrophils.

## 5 Conclusions

Since their discovery as part of the immune system, neutrophils have been viewed as the primary responders employing relatively primitive defensive mechanisms such as phagocytosis, production of ROS and release of NETs. Recent evidence is pointing out to the presence of well-defined signaling cascades resulting from the recognition of pathogenic molecular elements. The response of the neutrophils to presence of cytosolic DNA aids the immune response by magnifying the initial response to the pathogen. The release of IL-1β and type I IFNs can profoundly alter the profile of the downstream immune cells and influence the outcome of the immune response. Identification of other pathogen recognition pathways in the neutrophils will allow us to further understand the role of these key defenders of the immune system and their relevance in different diseases.

## Authorship

All named authors meet the International Committee of Medical Journal Editors (ICMJE) criteria for authorship for this article, take responsibility for the integrity of the work as a whole, and have given their approval for this version to be published.

## Author contributions

AM conceptualized and wrote the first draft of the manuscript, all authors provided significant intellectual input and reviewed, edited, and approved the final manuscript. All authors contributed to the article and approved the submitted version.

## References

[1] Burn GL Foti A Marsman G Patel DF Zychlinsky A. The neutrophil. Immunity (2021) 54(7):1377–91. doi: 10.1016/j.immuni.2021.06.006 34260886

[2] Hidalgo A Chilvers ER Summers C Koenderman L. The neutrophil life cycle. Trends Immunol (2019) 40(7):584–97. doi: 10.1016/j.it.2019.04.013 31153737

[3] Naish E Wood AJ Stewart AP Routledge M Morris AC Chilvers ER. The formation and function of the neutrophil phagosome. Immunol Rev (2023) 314(1):158–80. doi: 10.1111/imr.13173 PMC10952784 36440666

[4] Winterbourn CC Kettle AJ Hampton MB. Reactive oxygen species and neutrophil function. Annu Rev Biochem (2016) 85:765–92. doi: 10.1146/annurev-biochem-060815-014442 27050287

[5] Cowland JB Borregaard N. Granulopoiesis and granules of human neutrophils. Immunol Rev (2016) 273(1):11–28. doi: 10.1111/imr.12440 27558325

[6] Lee WL Harrison RE Grinstein S. Phagocytosis by neutrophils. Microbes Infect (2003) 5(14):1299–306. doi: 10.1016/j.micinf.2003.09.014 14613773

[7] Segal AW. How neutrophils kill microbes. Annu Rev Immunol (2005) 23:197–223. doi: 10.1146/annurev.immunol.23.021704.115653 15771570 PMC2092448

[8] Singhal A Kumar S. Neutrophil and remnant clearance in immunity and inflammation. Immunology (2022) 165(1):22–43. doi: 10.1111/imm.13423 34704249

[9] Lin A Lore K. Granulocytes: new members of the antigen-presenting cell family. Front Immunol (2017) 8:1781. doi: 10.3389/fimmu.2017.01781 29321780 PMC5732227

[10] Brinkmann V Reichard U Goosmann C Fauler B Uhlemann Y Weiss DS. Neutrophil extracellular traps kill bacteria. Science (2004) 303(5663):1532–5. doi: 10.1126/science.1092385 15001782

[11] Hidalgo A Libby P Soehnlein O Aramburu IV Papayannopoulos V Silvestre-Roig C. Neutrophil extracellular traps: from physiology to pathology. Cardiovasc Res (2022) 118(13):2737–53. doi: 10.1093/cvr/cvab329 PMC9586562 34648022

[12] Thiam HR Wong SL Wagner DD Waterman CM. Cellular mechanisms of NETosis. Annu Rev Cell Dev Biol (2020) 36:191–218. doi: 10.1146/annurev-cellbio-020520-111016 32663035 PMC8499668

[13] Papayannopoulos V. Neutrophil extracellular traps in immunity and disease. Nat Rev Immunol (2018) 18(2):134–47. doi: 10.1038/nri.2017.105 28990587

[14] Lood C Blanco LP Purmalek MM Carmona-Rivera C De Ravin SS Smith CK. Neutrophil extracellular traps enriched in oxidized mitochondrial DNA are interferogenic and contribute to lupus-like disease. Nat Med (2016) 22(2):146–53. doi: 10.1038/nm.4027 PMC4742415 26779811

[15] Kawai T Akira S. TLR signaling. Semin Immunol (2007) 19(1):24–32. doi: 10.1016/j.smim.2006.12.004 17275323

[16] Briard B Place DE Kanneganti TD. DNA Sensing in the innate immune response. Physiol (Bethesda) (2020) 35(2):112–24. doi: 10.1152/physiol.00022.2019 PMC7276919 32027562

[17] Mankan AK Schmidt T Chauhan D Goldeck M Honing K Gaidt M. Cytosolic RNA : DNA hybrids activate the cGAS-STING axis. EMBO J (2014) 33(24):2937–46. doi: 10.15252/embj.201488726 PMC4282641 25425575

[18] Hornung V Latz E. Intracellular DNA recognition. Nat Rev Immunol (2010) 10(2):123–30. doi: 10.1038/nri2690 20098460

[19] Kayagaki N Stowe IB Lee BL O’Rourke K Anderson K Warming S. Caspase-11 cleaves gasdermin d for non-canonical inflammasome signalling. Nature (2015) 526(7575):666–71. doi: 10.1038/nature15541 26375259

[20] Sun L Wu J Du F Chen X Chen ZJ. Cyclic GMP-AMP synthase is a cytosolic DNA sensor that activates the type I interferon pathway. Science (2013) 339(6121):786–91. doi: 10.1126/science.1232458 PMC3863629 23258413

[21] Decout A Katz JD Venkatraman S Ablasser A. The cGAS-STING pathway as a therapeutic target in inflammatory diseases. Nat Rev Immunol (2021) 21(9):548–69. doi: 10.1038/s41577-021-00524-z PMC8029610 33833439

[22] Samson N Ablasser A. The cGAS-STING pathway and cancer. Nat Cancer (2022) 3(12):1452–63. doi: 10.1038/s43018-022-00468-w 36510011

[23] Motwani M Pesiridis S Fitzgerald KA. DNA Sensing by the cGAS-STING pathway in health and disease. Nat Rev Genet (2019) 20(11):657–74. doi: 10.1038/s41576-019-0151-1 31358977

[24] Zhang X Kluger Y Nakayama Y Poddar R Whitney C DeTora A. Gene expression in mature neutrophils: early responses to inflammatory stimuli. J Leukoc Biol (2004) 75(2):358–72. doi: 10.1189/jlb.0903412 14634056

[25] Newburger PE Subrahmanyam YV Weissman SM. Global analysis of neutrophil gene expression. Curr Opin Hematol (2000) 7(1):16–20. doi: 10.1097/00062752-200001000-00004 10608499

[26] Hayashi F Means TK Luster AD. Toll-like receptors stimulate human neutrophil function. Blood (2003) 102(7):2660–9. doi: 10.1182/blood-2003-04-1078 12829592

[27] Lindau D Mussard J Rabsteyn A Ribon M Kotter I Igney A. TLR9 independent interferon alpha production by neutrophils on NETosis in response to circulating chromatin, a key lupus autoantigen. Ann Rheum Dis (2014) 73(12):2199–207. doi: 10.1136/annrheumdis-2012-203041 24013727

[28] Zhang Q Raoof M Chen Y Sumi Y Sursal T Junger W. Circulating mitochondrial DAMPs cause inflammatory responses to injury. Nature (2010) 464(7285):104–7. doi: 10.1038/nature08780 PMC2843437 20203610

[29] Mankan AK Dau T Jenne D Hornung V. The NLRP3/ASC/Caspase-1 axis regulates IL-1beta processing in neutrophils. Eur J Immunol (2012) 42(3):710–5. doi: 10.1002/eji.201141921 22213227

[30] Bakele M Joos M Burdi S Allgaier N Poschel S Fehrenbacher B. Localization and functionality of the inflammasome in neutrophils. J Biol Chem (2014) 289(8):5320–9. doi: 10.1074/jbc.M113.505636 PMC3931087 24398679

[31] Chauhan D Demon D Vande Walle L Paerewijck O Zecchin A Bosseler L. GSDMD drives canonical inflammasome-induced neutrophil pyroptosis and is dispensable for NETosis. EMBO Rep (2022) 23(10):e54277. doi: 10.15252/embr.202154277 35899491 PMC9535806

[32] Tamassia N Le Moigne V Rossato M Donini M McCartney S Calzetti F. Activation of an immunoregulatory and antiviral gene expression program in poly(I:C)-transfected human neutrophils. J Immunol (2008) 181(9):6563–73. doi: 10.4049/jimmunol.181.9.6563 18941247

[33] Xia P Wang S Ye B Du Y Huang G Zhu P. Sox2 functions as a sequence-specific DNA sensor in neutrophils to initiate innate immunity against microbial infection. Nat Immunol (2015) 16(4):366–75. doi: 10.1038/ni.3117 25729924

[34] Gupta S Nakabo S Blanco LP O’Neil LJ Wigerblad G Goel RR. Sex differences in neutrophil biology modulate response to type I interferons and immunometabolism. Proc Natl Acad Sci U.S.A. (2020) 117(28):16481–91. doi: 10.1073/pnas.2003603117 PMC7368314 32601182

[35] Yu Z Chen T Cao X. Neutrophil sensing of cytoplasmic, pathogenic DNA in a cGAS-STING-independent manner. Cell Mol Immunol (2016) 13(4):411–4. doi: 10.1038/cmi2015.34 PMC4947819 25914935

[36] Mankan AK Hornung V. Sox2 as a servant of two masters. Nat Immunol (2015) 16(4):335–6. doi: 10.1038/ni.3121 25789674

[37] Trevani AS Chorny A Salamone G Vermeulen M Gamberale R Schettini J. Bacterial DNA activates human neutrophils by a CpG-independent pathway. Eur J Immunol (2003) 33(11):3164–74. doi: 10.1002/eji.200324334 14579285

[38] Zhang Q Itagaki K Hauser CJ. Mitochondrial DNA is released by shock and activates neutrophils via p38 map kinase. Shock (2010) 34(1):55–9. doi: 10.1097/SHK.0b013e3181cd8c08 19997055

[39] Riley JS Tait SW. Mitochondrial DNA in inflammation and immunity. EMBO Rep (2020) 21(4):e49799. doi: 10.15252/embr.201949799 32202065 PMC7132203

[40] Zhang W Li G Luo R Lei J Song Y Wang B. Cytosolic escape of mitochondrial DNA triggers cGAS-STING-NLRP3 axis-dependent nucleus pulposus cell pyroptosis. Exp Mol Med (2022) 54(2):129–42. doi: 10.1038/s12276-022-00729-9 PMC8894389 35145201

[41] Kienle K Glaser KM Eickhoff S Mihlan M Knopper K Reategui E. Neutrophils self-limit swarming to contain bacterial growth in vivo. Science (2021) 372(6548). doi: 10.1126/science.abe7729 PMC8926156 34140358

[42] Leliefeld PH Koenderman L Pillay J. How neutrophils shape adaptive immune responses. Front Immunol (2015) 6:471. doi: 10.3389/fimmu.2015.00471 26441976 PMC4568410

[43] Mantovani A Cassatella MA Costantini C Jaillon S. Neutrophils in the activation and regulation of innate and adaptive immunity. Nat Rev Immunol (2011) 11(8):519–31. doi: 10.1038/nri3024 21785456

[44] Scapini P Cassatella MA. Social networking of human neutrophils within the immune system. Blood (2014) 124(5):710–9. doi: 10.1182/blood-2014-03-453217 24923297

[45] Li Y Wang W Yang F Xu Y Feng C Zhao Y. The regulatory roles of neutrophils in adaptive immunity. Cell Commun Signal (2019) 17(1):147. doi: 10.1186/s12964-019-0471-y 31727175 PMC6854633

[46] Apel F Andreeva L Knackstedt LS Streeck R Frese CK Goosmann C. The cytosolic DNA sensor cGAS recognizes neutrophil extracellular traps. Sci Signal (2021) 14(673). doi: 10.1126/scisignal.aax7942 33688080

[47] Granot Z Henke E Comen EA King TA Norton L Benezra R. Tumor entrained neutrophils inhibit seeding in the premetastatic lung. Cancer Cell (2011) 20(3):300–14. doi: 10.1016/j.ccr.2011.08.012 PMC3172582 21907922

[48] Benevides L da Fonseca DM Donate PB Tiezzi DG De Carvalho DD de Andrade JM. IL17 promotes mammary tumor progression by changing the behavior of tumor cells and eliciting tumorigenic neutrophils recruitment. Cancer Res (2015) 75(18):3788–99. doi: 10.1158/0008-5472.CAN-15-0054 PMC8101363 26208902

[49] Mizuno R Kawada K Itatani Y Ogawa R Kiyasu Y Sakai Y. The role of tumor-associated neutrophils in colorectal cancer. Int J Mol Sci (2019) 20(3). doi: 10.3390/ijms20030529 PMC6386937 30691207

[50] Kalafati L Mitroulis I Verginis P Chavakis T Kourtzelis I. Neutrophils as orchestrators in tumor development and metastasis formation. Front Oncol (2020) 10:581457. doi: 10.3389/fonc.2020.581457 33363012 PMC7758500

[51] Mantovani A Dinarello CA Molgora M Garlanda C. Interleukin-1 and related cytokines in the regulation of inflammation and immunity. Immunity (2019) 50(4):778–95. doi: 10.1016/j.immuni.2019.03.012 PMC7174020 30995499

[52] McNab F Mayer-Barber K Sher A Wack A O’Garra A. Type I interferons in infectious disease. Nat Rev Immunol (2015) 15(2):87–103. doi: 10.1038/nri3787 25614319 PMC7162685

[53] Ito T Amakawa R Inaba M Ikehara S Inaba K Fukuhara S. Differential regulation of human blood dendritic cell subsets by IFNs. J Immunol (2001) 166(5):2961–9. doi: 10.4049/jimmunol.166.5.2961 11207245

[54] Man SM Karki R Kanneganti TD. AIM2 inflammasome in infection, cancer, and autoimmunity: role in DNA sensing, inflammation, and innate immunity. Eur J Immunol (2016) 46(2):269–80. doi: 10.1002/eji.201545839 PMC4758349 26626159

[55] Liu N Pang X Zhang H Ji P. The cGAS-STING pathway in bacterial infection and bacterial immunity. Front Immunol (2021) 12:814709. doi: 10.3389/fimmu.2021.814709 35095914 PMC8793285

[56] Kwon J Bakhoum SF. The cytosolic DNA-sensing cGAS-STING pathway in cancer. Cancer Discovery (2020) 10(1):26–39. doi: 10.1158/2159-8290.CD-19-0761 31852718 PMC7151642

[57] Witter AR Okunnu BM Berg RE. The essential role of neutrophils during infection with the intracellular bacterial pathogen listeria monocytogenes. J Immunol (2016) 197(5):1557–65. doi: 10.4049/jimmunol.1600599 PMC4995063 27543669

[58] Li J Bakhoum SF. The pleiotropic roles of cGAS-STING signaling in the tumor microenvironment. J Mol Cell Biol (2022) 14(4). doi: 10.1093/jmcb/mjac019 PMC9354322 35325182

[59] Chiang CC Cheng WJ Korinek M Lin CY Hwang TL. Neutrophils in psoriasis. Front Immunol (2019) 10:2376. doi: 10.3389/fimmu.2019.02376 31649677 PMC6794444

[60] Mrowietz U. Neutrophils’ sexiness is independent of trendy fashion. Exp Dermatol (2017) 26(4):312–3. doi: 10.1111/exd.13102 27248359

[61] Sadeghi M Dehnavi S Jamialahmadi T Johnston TP Sahebkar A. Neutrophil extracellular trap: a key player in the pathogenesis of autoimmune diseases. Int Immunopharmacol (2023) 116:109843. doi: 10.1016/j.intimp.2023.109843 36764274

[62] Toichi E Tachibana T Furukawa F. Rapid improvement of psoriasis vulgaris during drug-induced agranulocytosis. J Am Acad Dermatol (2000) 43(2 Pt 2):391–5. doi: 10.1067/mjd.2000.103264 10901732

[63] Mizutani Y Fujii K Kawamura M Inoue M Mizutani YH Matsuyama K. Intensive granulocyte and monocyte adsorption apheresis for generalized pustular psoriasis. J Dermatol (2020) 47(11):1326–9. doi: 10.1111/1346-8138.15569 32860246

[64] Lin AM Rubin CJ Khandpur R Wang JY Riblett M Yalavarthi S. Mast cells and neutrophils release IL-17 through extracellular trap formation in psoriasis. J Immunol (2011) 187(1):490–500. doi: 10.4049/jimmunol.1100123 21606249 PMC3119764

[65] Mankan AK Canli O Schwitalla S Ziegler P Tschopp J Korn T. TNF-alpha-dependent loss of IKKbeta-deficient myeloid progenitors triggers a cytokine loop culminating in granulocytosis. Proc Natl Acad Sci U.S.A. (2011) 108(16):6567–72. doi: 10.1073/pnas.1018331108 PMC3080999 21464320

[66] Luo Y Hara T Kawashima A Ishido Y Suzuki S Ishii N. Pathological role of excessive DNA as a trigger of keratinocyte proliferation in psoriasis. Clin Exp Immunol (2020) 202(1):1–10. doi: 10.1111/cei.13455 32415989 PMC7586253

[67] Li C Liu W Wang F Hayashi T Mizuno K Hattori S. DNA Damage-triggered activation of cGAS-STING pathway induces apoptosis in human keratinocyte HaCaT cells. Mol Immunol (2021) 131:180–90. doi: 10.1016/j.molimm.2020.12.037 33423764

[68] Zhang Y Xu X Cheng H Zhou F. AIM2 and psoriasis. Front Immunol (2023) 14:1085448. doi: 10.3389/fimmu.2023.1085448 36742336 PMC9889639

[69] Kaur A Goggolidou P. Ulcerative colitis: understanding its cellular pathology could provide insights into novel therapies. J Inflammation (2020) 17(1):15. doi: 10.1186/s12950-020-00246-4 PMC7175540 32336953

[70] Kurimoto N Nishida Y Hosomi S Itani S Kobayashi Y Nakata R. Neutrophil-to-lymphocyte ratio may predict clinical relapse in ulcerative colitis patients with mucosal healing. PloS One (2023) 18(1):e0280252. doi: 10.1371/journal.pone.0280252 36634124 PMC9836288

[71] Bennike TB Carlsen TG Ellingsen T Bonderup OK Glerup H Bøgsted M. Neutrophil extracellular traps in ulcerative colitis: a proteome analysis of intestinal biopsies. Inflammatory Bowel Dis (2015) 21(9):2052–67. doi: 10.1097/MIB.0000000000000460 PMC4603666 25993694

[72] Dinallo V Marafini I Di Fusco D Laudisi F Franzè E Di Grazia A. Neutrophil extracellular traps sustain inflammatory signals in ulcerative colitis. J Crohn’s Colitis (2019) 13(6):772–84. doi: 10.1093/ecco-jcc/jjy215 30715224

[73] Pai RK Hartman DJ Rivers CR Regueiro M Schwartz M Binion DG. Complete resolution of mucosal neutrophils associates with improved long-term clinical outcomes of patients with ulcerative colitis. Clin Gastroenterol Hepatol (2020) 18(11):2510–2517.e5. doi: 10.1016/j.cgh.2019.12.011 31843598

[74] Tanaka T Okanobu H Yoshimi S Murakami E Kogame A Imagawa H. In patients with ulcerative colitis, adsorptive depletion of granulocytes and monocytes impacts mucosal level of neutrophils and clinically is most effective in steroid naïve patients. Digestive Liver Dis (2008) 40(9):731–6. doi: 10.1016/j.dld.2008.02.012 18387860

[75] Flood P Fanning A Woznicki JA Crowley T Christopher A Vaccaro A. DNA Sensor-associated type I interferon signaling is increased in ulcerative colitis and induces JAK-dependent inflammatory cell death in colonic organoids. Am J Physiol Gastrointest Liver Physiol (2022) 323(5):G439–g460. doi: 10.1152/ajpgi.00104.2022 36165492

[76] Kwantwi LB. Overcoming anti-PD-1/PD-L1 immune checkpoint blockade resistance: the role of macrophage, neutrophils and mast cells in the tumor microenvironment. Clin Exp Med (2023). doi: 10.1007/s10238-023-01059-4 37022584

[77] Que H Fu Q Lan T Tian X Wei X. Tumor-associated neutrophils and neutrophil-targeted cancer therapies. Biochim Biophys Acta Rev Cancer (2022) 1877(5):188762. doi: 10.1016/j.bbcan.2022.188762 35853517

[78] Sharma BR Karki R Kanneganti TD. Role of AIM2 inflammasome in inflammatory diseases, cancer and infection. Eur J Immunol (2019) 49(11):1998–2011. doi: 10.1002/eji.201848070 31372985 PMC7015662

[79] Di Micco A Frera G Lugrin J Jamilloux Y Hsu ET Tardivel A. AIM2 inflammasome is activated by pharmacological disruption of nuclear envelope integrity. Proc Natl Acad Sci U.S.A. (2016) 113(32):E4671–80. doi: 10.1073/pnas.1602419113 PMC4987819 27462105

[80] Novak D Huser L Elton JJ Umansky V Altevogt P Utikal J. SOX2 in development and cancer biology. Semin Cancer Biol (2020) 67(Pt 1):74–82. doi: 10.1016/j.semcancer.2019.08.007 31412296

[81] Zhang S Xiong X Sun Y. Functional characterization of SOX2 as an anticancer target. Signal Transduct Target Ther (2020) 5(1):135. doi: 10.1038/s41392-020-00242-3 32728033 PMC7391717

[82] Grimm D Bauer J Wise P Kruger M Simonsen U Wehland M. The role of SOX family members in solid tumours and metastasis. Semin Cancer Biol (2020) 67(Pt 1):122–53. doi: 10.1016/j.semcancer.2019.03.004 30914279

[83] Zhu Y Huang S Chen S Chen J Wang Z Wang Y. SOX2 promotes chemoresistance, cancer stem cells properties, and epithelial-mesenchymal transition by beta-catenin and Beclin1/autophagy signaling in colorectal cancer. Cell Death Dis (2021) 12(5):449. 33953166 10.1038/s41419-021-03733-5 PMC8100126

